# How does the climate risk affect the firm growth: Evidence from China

**DOI:** 10.1371/journal.pone.0343426

**Published:** 2026-02-25

**Authors:** Yanxue Zhang, Zirong Li

**Affiliations:** 1 School of Economics and Business Administration, Yibin University, Yibin, Sichuan, China; 2 Key Laboratory for Digital Analysis of Urban-Rural Industrial Integration Development and Intelligent Decision Making, Yibin, Sichuan, China; Guilin University of Aerospace Technology, CHINA

## Abstract

Corporate development is threatened by climate risk, which challenges business operations and long-term planning at an unprecedented scale and frequency. Based on the challenge–threat theory framework, this study uses data from Chinese A-share listed companies from 2008 to 2022 to systematically explore the effect of climate risk on firm growth and its transmission mechanisms. The findings indicate that (1) climate risk exhibits an inverted U-shaped non-linear relationship with firm growth. Moderate climate risk promotes firm growth by stimulating adaptive strategies (e.g., technological upgrades and financing expansion), but once the threshold is exceeded, resource constraints intensify, leading to a significant decline in growth potential. (2) Climate risk influences internal control quality and operational cost control efficiency through managers’ cognitive assessments (challenges or threats), which, in turn, indirectly affect the potential for firm growth by reducing agency costs, improving earnings quality, expanding profit margins, and lowering credit risks. (3) The level of financial development moderates the inflection point of climate risk. In financially developed regions, firms have greater access to credit resources, and managers maintain a more persistent perception of climate risk as a challenge. Their inverted U-shaped inflection point is significantly higher than that of firms in less developed regions, enabling them to view climate risk as a long-term challenge and sustain its promotional effect. This study integrates macro- and micro-perspectives to reveal the double-edged sword effect of climate risk. The theoretical foundation and practical insights can help enterprises respond to climate risk dynamically and formulate differentiated policies.

## 1. Introduction

Enterprises are closely connected to the external environment, and their business activities are strongly affected by climate and environmental changes. The rapid development of the Chinese economy has made resource scarcity and environmental pollution major obstacles to high-quality development. Climate risks, such as extreme weather, challenge daily operations and long-term planning at an unprecedented scale and frequency and directly affect business development. Two questions are pertinent here: Does climate risk affect business growth? If so, what mechanisms are involved? Answering these questions should help enterprises to better recognise and understand the challenges posed by climate change and take effective measures to prevent and respond to them to safeguard their long-term stability in development. Revealing the mechanism underlying the effect of climate risk on enterprise growth further helps enterprises to better participate in the fight against climate change. Overall, these developments should enhance society’s ability to cope with climate change and address the challenges of global climate change.

Literature closely related to this study is the effect of climate risk. On the macro-side, studies on the effect of climate risk are mainly related to health, economy, social relations, and population [1]. For example, extreme weather events have long-lasting negative effects on economic growth, with labour supply and productivity being significantly affected by extreme weather, especially in outdoor labour-intensive industries. Energy systems are particularly sensitive to extreme changes in temperature, putting pressure on the energy supply and transmission. At the micro-level, some scholars believe that climate change risks bring new investment and market opportunities, encourage corporations to take on environmental social responsibility [2], and enhance corporate sustainability performance. Climate risk may also negatively affect business development by disrupting supply chains [3], increasing insurance premiums [4] and operating costs, and affecting corporate financing constraints, profitability, and leverage [5].

Our study is also relevant to the literature on the factors affecting firm growth. Firm growth and its influencing factors are complex. Firm growth is influenced by both external and internal factors. In terms of the external macro-environment, government policies [6], financial and tax systems [7], and social service systems have profound effects on enterprises’ growth potential. Internal micro-factors can be further divided into two categories: financial and non-financial. Financial indicators include asset turnover [8], gross profit margin, debt ratio, and growth rate of the main business income. Non-financial indicators include various resources within the enterprise, knowledge, innovation capacity [9], managerial quality [8], and other aspects. In recent years, research has begun to examine the role of managerial cognition in the transmission of climate risk. Managers’ perceptions of climate risk directly affect strategic decisions and resource allocation. For example, managerial attention to climate risk significantly influences the proactivity of corporate responses [10], while CEOs’ climate risk perception bias may further transmit to firms’ choices of debt structure [11]. However, the existing literature still mostly focuses on the negative economic consequences of climate risk and the mechanisms for coping with them, and lacks a systematic theoretical framework to explain how the objective external pressure, through managers’ micro-level cognitive appraisal, ultimately yields complex effects on firms.

Based on the challenge–threat framework, this paper examines how city-level climate risk affects firm growth through the micro-psychological channel of executives’ cognitive appraisal. This provides a fresh perspective between climate risk and corporate sustainable development. Using data on Chinese listed firms from 2008 to 2022, we employ a two-way fixed effects panel model. We find an inverted U-shaped relationship between climate risk and firm growth, which stems from managers’ differential risk appraisal. This appraisal process subsequently influences growth via two channels: internal control quality and operating cost control. Furthermore, regional financial development significantly moderates the turning point of this relationship.

The possible marginal contributions of this paper are as follows: (1) It goes beyond the literature’s common focus on the negative consequences of climate risk [1,3–5]. It not only confirms the dampening effect when climate risk is too high, but also shows that moderate risk can have a positive effect by motivating firms to adapt and adjust, thereby deepening understanding of the complexity of the microeconomic consequences of climate risk. (2) It introduces the challenge–threat framework and formalises the managerial cognitive appraisal mechanism, offering a novel theoretical perspective on how external environmental pressure is transformed into internal strategic responses, and it extends the research frontier on the drivers of firm growth.

The remaining paper is organised as follows. Section 2 presents the research hypotheses. Section 3 describes the data and variables. Section 4 analyses the empirical results. Section 5 investigates the mechanisms. Section 6 tests for heterogeneity. Finally, the entire study is discussed and summarized in Section 7.

## 2. Theories and hypotheses

### 2.1. Challenge–threat theory

Individuals who face stressful situations display different psychological and behavioural responses based on their comprehensive assessment of the situation. In 1987, Lazarus and Folkman [12] proposed the cognitive appraisal theory of stress, which emphasises that an individual’s cognitive appraisal of a stressor is not only affected by the nature of the stressor but also depends on the richness of the resources at the individual’s disposal. Lazarus and Folkman further developed the challenge–threat theory, which has since gained widespread theoretical acceptance in discussions on the effect of social stress on individual decision-making. The challenge–threat theory states that individuals may exhibit different responses to resilience (i.e., challenge) or vulnerability (i.e., threat) when faced with potential stress [13].

An individual’s mental processes in the face of stress involve two core assessment stages: need and resource assessment [14]. Needs assessment evaluates the amount of resources required to cope with stress in terms of effort, uncertainty, and risk. Resource assessment is a comprehensive evaluation of an individual’s current resources [15]. Based on the results of these two assessments, individuals perceive stress as a challenge when the resources they hold meet or slightly exceed the resources needed to cope with stress and vice versa [16,17]. Follow-up research further suggests that, even if the resources an individual possesses are slightly less than what is needed, the individual may still perceive stress as a challenge as long as the gap is not large. It is only when the resources are significantly less that they are perceived as threats [17]. Challenges and threats are important predictors of future performance [18]. A large corpus of literature suggests that challenges facilitate decision-making and may enhance performance, whereas threats do not [14,19].

In summary, according to the challenge–threat theory, individuals facing stress undergo an integrated psychological process comprising two dimensions: need assessment and resource assessment. This appraisal mechanism is moderated by factors such as firm size, regional financial development, and government support [20,21]. Based on the results of the assessment, stress is evaluated as either a challenge or a threat, with the former favouring better performance and the latter the opposite [22].

### 2.2. Climate risk, challenge assessment, and firm growth

As an open system, an organisation’s growth depends on its resource interactions with the external environment. In the production and business operations of enterprises, capital, labour, land, and technological innovation constitute the most critical production factor system. Climate risk affects managers’ challenge/threat assessments by altering the availability of core production factors, such as labour, capital, and technology, thereby influencing firm growth. Under moderate climate risk, a company’s redundant resources—that is, those that have not yet been fully utilized—can serve as a buffer [23] and be reallocated when environmental changes occur, providing firms with flexibility to adapt to uncertain and dynamic environments. At this stage, resource pressure has not yet exceeded the resources available to the firm, and managers tend to assess them as challenges.

First, within a moderate range of climate risk, the fundamental functions of the labour market are not undermined. Instead, firm-level human capital allocation can be optimised through the dual mechanisms of talent retention and structural upgrading. First, the compensating wage differential mechanism promotes stability in the overall talent pool. Based on Roback’s spatial equilibrium theory, employees make a trade-off between the loss of environmental quality and monetary compensation [24,25]; when a wage premium is sufficient to offset climate risk, the labour market can maintain a dynamic equilibrium [26]. In the Chinese context, the Hukou (household registration) system and a cultural attachment to one’s homeland further constrain the outflow of labour in low-risk scenarios, enabling firms to maintain personnel stability and prevent irrational attrition through wage compensation. Second, moderate climate risk drives the reallocation of labour across sectors and regions. Research indicates that climate risk reduces productivity in sectors such as agriculture, compelling labour to migrate toward other sectors and regions with a comparative advantage [27]. This structural adjustment, in turn, compels firms to optimise their internal human capital structure by eliminating inefficient positions and strengthening the cultivation and retention of high-skilled talent. Under the combined influence of these mechanisms, not only do managers perceive their existing human resources as sufficient to cope with current risks, but they also become more willing to undertake forward-looking human capital investment. For instance, by providing training to enhance employees’ climate adaptation skills and their willingness to engage in a green transition [28] firms can transform potential climate challenges into opportunities for organisational development.

Second, moderate climate risk can induce firms to optimise the allocation of credit resources. First, when climate risk is moderate (i.e., relatively low), the external business environment is more stable and exhibits lower uncertainty, meaning firms face a more favourable financing environment [29] and lower financing costs. Second, financial institutions, as market entities directly affected by climate risk, will proactively adjust their business strategies and promote a green transition [30,31], for example by developing green credit products [32] and strengthening environmental information disclosure requirements. This not only fosters a green transition for both banks and firms but also prevents credit crunches that could arise from excessive risk aversion. Furthermore, when firms successfully implement climate change mitigation and adaptation strategies, their strong climate performance can significantly enhance their reputation, sending positive signals to investors and financial institutions [33]. This allows them to secure more favourable financing terms [34] and establish a financing cost advantage [35]. Moreover, government subsidies or guarantee mechanisms for green projects help to reduce banks’ credit risk [36], incentivising them to offer loans to relevant firms at lower interest rates. In a moderate climate risk scenario, the coordinated alignment of external policies and market instruments can guide financial resources to flow more effectively towards climate risk-related areas, thus transforming moderate external climate pressure into a stable opportunity for firms to secure specialised financing and support long-term development.

Moderate climate risk also stimulates corporate technological innovation through multiple channels [37]. From an external perspective, the environmental pressure brought about by climate risk will push the government to strengthen environmental regulations, forcing companies to increase their R&D investment in areas such as energy-saving technologies and green production processes [38] in order to meet higher environmental standards. The increased market demand for climate-adaptive products (e.g., heat-resistant materials and energy-saving equipment) and mitigation technologies (e.g., low-carbon energy technologies) creates market incentives for corporate technological innovation. Companies can proactively integrate climate risk responses into their production operations, resource allocation, and risk management processes, thereby enhancing technological innovation through increased automation and adjustment to supply chain strategies [39]. Technological innovation enhances firms’ core capabilities and resilience, and significantly drives their growth.

### 2.3. Climate risk, threat assessment, and firm growth

As climate risk intensifies, the resources required to address it will exceed enterprises’ existing resource capacity. Managers then face increased pressure on resources such as labour, capital, and technology, which leads them to view climate risk as a threat.

Conversely, rising climate risk increases the weight that employees place on non-monetary benefits, such as health and environmental quality, potentially causing these factors to outweigh the appeal of monetary compensation. This exacerbates talent attrition for firms and particularly elevates the risk of losing high-skilled labour. Good air quality is considered an important non-monetary benefit [25], and employees trade off environmental quality against wages [25]. When the health costs associated with a deteriorating climate exceed the benefits of employment, labour reallocation occurs [40]. High-skilled labour, being more competitive and possessing greater job opportunities and mobility, is particularly sensitive to climatic conditions. Once extreme weather threatens their health, they are more likely to proactively relocate to regions and firms with better climatic conditions rather than merely seeking wage compensation. This leads to firms not only losing key talent but also incurring substantial additional costs for recruitment and training. Consequently, team productivity and stability are significantly impacted, which in turn intensifies management’s perception of climate risk as a threat.

As climate risk intensifies, the financial environment for firms deteriorates, characterized mainly by a contraction in financing channels and an increase in financing costs. First, extreme weather events can directly damage corporate assets, weaken debt-servicing capacity, and raise default risk [41]. At the same time, deposit withdrawals for post-disaster recovery further reduce credit supply [42]. Driven by risk aversion, banks tighten credit standards [43], adopt more prudent strategies [44], cut overall lending [45], and reduce credit support to high-risk regions and industries while curtailing long-term finance due to exposure concerns [46,47]. Second, the narrowing of financing channels raises corporate financing costs. In a context of reduced credit, loans still available carry stricter terms such as higher interest rates, tighter collateral requirements, and stricter covenants, significantly increasing the cost of debt. Meanwhile, investors in capital markets price in climate risk by demanding a higher risk premium for firms with greater cash-flow uncertainty [48], which raises the cost of equity. Overall, the broad tightening of financing channels and the marked rise in financing costs reinforce management’s perception of climate risk as a threat.

Third, amplified climate risk suppresses corporate technological innovation through three primary channels: resource displacement, heightened uncertainty-induced barriers, and increased risks of employee attrition. Natural resource theory holds that companies will inevitably incur corresponding expense-based expenditures and capitalised expenditures to address expanded climate risk, thereby reducing their available resources and technological investments, which, in turn, will affect their technological innovation capabilities [39]. The uncertainty caused by increased climate risk further hinders corporate innovation. According to financial slack theory, companies in a state of financial slack are more conducive to engaging in technological innovation activities [49]. To address the increased uncertainty caused by expanding climate risk, companies may increase their precautionary cash holdings, thereby reducing the funds available for R&D, leading to a decline in innovation investment and suppressing the company’s R&D capabilities. Meanwhile, as climate risk intensifies, the employee turnover risk increases, which suppresses corporate technological innovation.

### 2.4. Summary

Drawing on the preceding analysis, we posit that the impact of climate risk on firm growth exhibits a non-linear inverted U-shaped relationship, being initially promotional and subsequently inhibitory. The core underlying mechanism for this relationship stems from management’s cognitive appraisal of climate risk. This is not a simple binary judgement but rather a continuous process based on the dynamic balance between a firm’s available resources and the demands imposed by the risk. This process, characterised by a ‘challenge-threat’ continuum of appraisal, drives differential strategic decisions and resource allocation behaviours. Consequently, the stimulative effect of climate risk on firm growth under a ‘challenge’ appraisal, followed by its inhibitory effect under a ‘threat’ appraisal, generates an inverted U-shaped curve.

When the intensity of climate risk is low, the corresponding demands for coping are small, and the firm’s existing resource buffers, such as human capital, financial reserves, and technological capabilities, appear relatively ample. Through their appraisal, managers conclude that existing resources are sufficient to manage the issues posed by the risk and thus perceive it as a ‘challenge’. In this state, managers adopt a proactive mindset, favouring exploratory innovation and strategic adjustments, such as increasing investment in green technology R&D and optimising human resource allocation [28]. These actions activate and reorganise corporate resources, fostering continuous improvement in firm growth and constituting the upward-sloping portion of the inverted U-shaped curve.

However, as the intensity of climate risk progressively increases, the demand for coping—arising from the need to repair physical damage and meet transitional compliance requirements— rises sharply, beginning to continuously erode the firm’s resource buffers. Defensive expenditures crowd out core resources that could be used for development. Consequently, management’s perception gradually shifts from ‘challenge’ to ‘threat’. Their strategic focus also pivots from proactive initiatives to risk prevention, leading to an increasing number of resources becoming immobilised in defensive assets such as insurance and emergency reserves. This crowds out investments that would otherwise be directed towards production expansion and innovative activities. This process continuously weakens the firm’s growth drivers, leading to a decline in its growth potential and forming the downward-sloping portion of the inverted U-shaped curve.

The inflection point connecting the rising and falling portions of the curve theoretically corresponds to the critical equilibrium at which a firm’s available resources match the demands of coping with risk. At this juncture, a fundamental shift in managerial cognition occurs. The precise location of this inflection point is not fixed; it is moderated by internal and external factors that influence the firm’s resource buffering capacity and risk response costs. Internally, stronger financial health and innovative capabilities and, externally, more developed financial markets and more robust policy support can enhance a firm’s resource buffer in a high-risk environment. This enables management to maintain a ‘challenge’ appraisal even at higher objective levels of climate risk, thereby prolonging the promotional phase of firm growth. This ultimately manifests as a shift in the inflection point towards a higher level of risk intensity.

**Fig 1:**
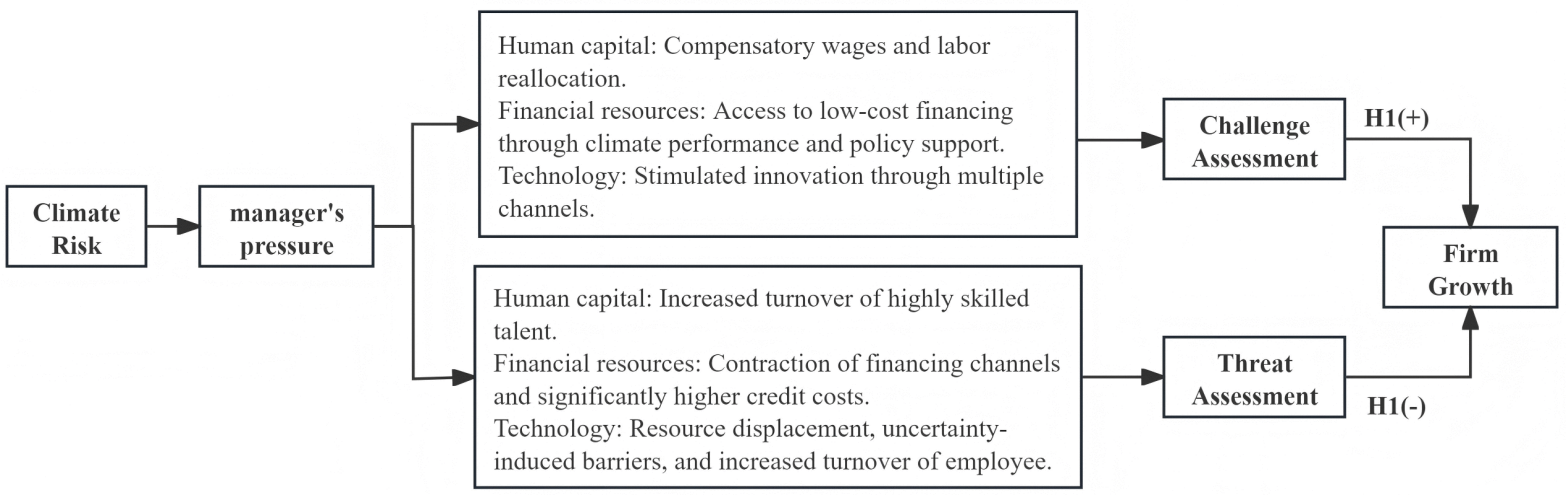
The relationship between climate risk and firm growth.

In summary, by influencing management’s continuous cognitive appraisal based on the resource-demand balance, climate risk triggers a sequential response that shifts from resource activation to resource crowding-out, ultimately resulting in an inverted U-shaped relationship with firm growth (Fig 1).

We thus propose that—

HI: An inverted-U-shaped non-linear relationship exists between climate risk and firm growth.

## 3. Method

### 3.1. Model design

To test the relationship between climate risk and firm growth, we established the following model:


$${GROW}_{it}\;=\;\alpha_{\text{0}}\;+\;\beta_{\text{1}}\times {CRSK}_{it}\;+\;\beta_{\text{2}}\times {CRSK\text{2}}_{it}\;+\;\eta \times X\;+\;\alpha_{i}\;+\;\lambda_{t}\;+\;\epsilon_{it}.$$ (1)

*GROW*_*it*_ represents the growth of firm *i* in year *t*. *CRSK*_*i*,*t*_ measures climate risk in the headquarters city of firm *i* in year *t*, *β*_*1*_ is its coefficient, *CRSK2*_*i*,*t*_ is the quadratic term of *CRSK*_*i*,*t*_, and *β*_*2*_ is its coefficient. A significantly negative *β*_*2*_ indicates an inverted U-shaped relationship between climate risk and firm growth. *X* denotes the control variable. $\alpha_{i}$ represents the individual fixed effect. $\lambda_{t}$ represents the time fixed effect. $\varepsilon_{i,t}$ represents the random error term.

### 3.2. Sample selection and data source

In June 2007, China issued the *National Programme for Responding to Climate Change*, which marked the nation’s first comprehensive policy document addressing climate change and initiated a structured effort to tackle climate risk in Chinese cities. Following the methodology of Chen et al. (2023) [20], we used all Chinese A-share listed companies on the Shanghai and Shenzhen stock exchanges from 2008 to 2022 as our initial corporate sample. In line with common practice in the literature [50,51], we employed a headquarters-based geographical matching approach to align firm-level data with city-level climate risk indicators. Specifically, we obtained the city code where each firm’s annual headquarters is located, matched it with the corresponding city-level climate risk indicators, and examined the its impact on firm growth.

The data were processed as follows: (1) samples with ST (special treatment, which means listed companies with negative net profit for two consecutive fiscal years) or PT (particular transfer, which means listed companies that stopped any transactions, cleared the price, and waited to be delisted) status were excluded; (2) all financial enterprises were excluded; (3) missing samples were excluded; (4) samples with only one observation were excluded; and (5) samples with operating costs or current assets less than 0 were excluded; (6)To mitigate the influence of outliers and improve distributional properties, natural logarithmic transformations were applied to continuous variables with right-skewed distributions. Additionally, we implemented a 1% Winsorised tail-trimming treatment for other continuous variables. Ultimately, 35,668 observations were obtained. We have deposited the minimally sufficient dataset and the Stata analysis code required to replicate our findings in Figshare (https://doi.org/10.6084/m9.figshare.31047769).

The data used to construct the CSRK were sourced from the statistics bureaus of each city; Patsnap (www.zhihuiya.com), data of which were sourced from the National Intellectual Property Administration; and Shanghai Dazhihui Caihui Data Technology Co., Ltd. (www.qyyjt.cn), the data of which were collected using web scraping technology from various administrative agencies in China. Carbon dioxide emissions for each city were sourced from the CEADs database (www.ceads.net). Corporate data were sourced from Wind Information (https://www.wind.com.cn). Urban-level control variables, such as *FSIZ* and *PGDP,* are sourced from the *City Statistical Yearbook*.

### 3.3. Variables

#### 3.3.1. Dependent variables.

The dependent variable is firm growth (*GROW*). We used the total asset growth rate to measure firm growth [21,52]. Following Lü et al. [53], the primary business revenue growth rate was used as a substitute variable (*rGROW*) for robustness testing.

#### 3.3.2. Independent variables.

The climate risk, the independent variable, is a comprehensive index composed of 10 city-level indicators (S1 Table).

The selected indicators are intended to capture multiple dimensions of climate change that are relevant to urban environments. First, climate risk originates from global warming, primarily driven by anthropogenic greenhouse gas emissions, and is significantly influenced by carbon dioxide. Besides, sulfur dioxide and industrial dust emissions are also linked to climate change. Second, economic activity can produce greenhouse gas emissions [54]. The total number of enterprises and the scale of registered capital represent the scale of economic activity, which correlates positively with the emission of pollutants. Third, technological innovation is a key response to climate risk. The patents classified as Y02 are green technologies and applications for mitigating or adapting to climate change in the patent classification catalog jointly issued by the European Patent Office and the U.S. Patent Office [55]. We employed the number and share of both applications and grants for such patents to capture innovative activity of each city. A higher volume of such patents indicates stronger technological readiness and provides a broader set of tools for climate adaptation and mitigation, thereby helping to lower operational and transitional uncertainties. Finally, climate risk manifests in environmental quality, particularly air quality [56]. Better air quality means that firms are less exposed to immediate climate-related hazards such as pollution. It also tends to signal more rigorous and effective environmental governance, which reduces short-term physical and policy risks for businesses.

Therefore, we selected 10 indicators – carbon dioxide emissions, sulphur dioxide emissions, industrial dust emissions, total number of enterprises, total registered capital of enterprises, number of patent applications for climate-friendly technologies and their proportion of all patent applications, number of granted patents for climate-friendly technologies and their proportion of all granted patents, and air quality excellence rate – to construct an initial index Clirsk using factor analysis.

The Kaiser–Meyer–Olkin (KMO) and Bartlett’s spherical tests are prerequisites for the factor analysis method. In calculating Clirsk, the KMO test showed that KMO = 0.737, which was greater than 0.6. The chi-square statistic of Bartlett’s test was 20566.111, with p-value <0.0001. Therefore, the 10 items met the prerequisites of the factor analysis.

The factor loadings of the rotated component matrix for the 10 indicators, based on the criterion of eigenvalues greater than 1, were extracted into four factors. Their variance contribution rates were 34.56%, 16.24%, 14.87%, and 10.09%, respectively, with a cumulative variance contribution rate of 75.75% (S2 Table), indicating that these four factors captured over 70% of the common variance in the data and possessed strong explanatory power. Then, using the variance contribution rates of the four factors as weights, a weighted sum of the four factors was calculated to obtain the initial composite index of climate risk (C*lirisk*). The C*lirisk* was divided by 100 to obtain the new variable CRSK, which served as a proxy variable for climate risk. The larger the value of CRSK, the higher the climate risk.

**Fig 2:**
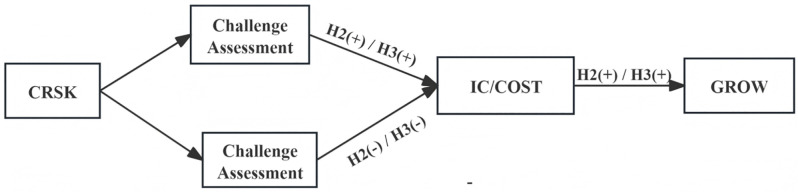
Trends of cimate risk in China from 2008 to 2022.

Using the available data, we calculated for 265 cities in mainland China from 2008 to 2022. We further computed three derived values of *Clirsk*: mean, median and weighted by city gross domestic product (*GDP*) (Fig 2). Fig 2 reveals an overall declining trend in climate risk, with the rate of decline accelerating after 2020.

**Fig 3:**
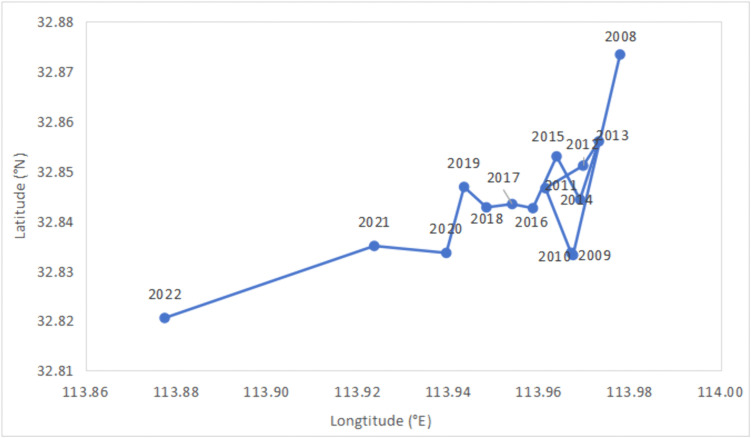
Gravity center migration path of climate risk in China from 2008 to 2022.

Meanwhile, we used ArcGIS (version 10.8) spatial analysis tools to measure the gravity center migration path of Clirsk (Fig 3). It shows that the gravity center remained relatively stable, with the overall direction of movement showing a zigzag trend toward the southwest, indicating that climate risk in China’s southwest region is increasing. In particular, the migration path of the gravity center toward the southwest in 2022 was significant, possibly owing to industrial relocation, leading to faster emission growth than those in other regions.

In selecting the replacement variables for CRSK, we adopted the method from Wang et al. (2023) [57] by constructing the alternative variable *rCRSK* using the natural logarithm transformation *ln(1 + Clirisk)*.

#### 3.3.3. Control variables.

To overcome the effect of omitted variables as much as possible, we controlled for a series of micro- and macro- variables that may affect the growth of enterprises (Table 1), with reference to the literature [21,52,53]. At the micro-level, these include the firm size (SIZE), leverage ratio (LEV), firm age (AGE), shareholding concentration (FIST), financing constraints (FINC), proportion of independent directors (INDR), board size (BSIZ), and CEO duality (DUAL). At the external macro-level, these include the per capita GDP of the city where the company’s headquarters is located (PGDP) and the level of city financial development (FSIZ). Time (Year) and individual dummy variables were introduced to control for time and individual effects.

**Table 1:** Description of variables.

Type of variable	Variable name	Variable symbol	Variable definition and caculation	Unit	Coverage	Data source	Access date
Dependent variables	Firm growth	*GROW*	Total asset growth rate	%	(2008–2022) Enterprise-level	Wind (https://www.wind.com.cn)	2024/06
		*rGROW*	(The growth rate of primary business revenue)/100	%			
Independent variables	Climate risk	*CRSK*	*Clirsk/100,* where *Clirisk* is derived from factor analysis based on 10 indicators.	_	(2008–2022) Urban-level	*Original index, calculated by the authors. (*see 3.3.2 Independent Variables)	2024/09
		*CRSK2*	*CRSK* squared	_			
		*rCRSK*	Ln(1 + C*lirisk)*	_			
		*rCRSK2*	*rCRSK* squared	_			
Intermediary variables	Cost control	*COST*	-(Operating costs/operating income)	%	(2008–2022) Enterprise-level	Wind (https://www.wind.com.cn)	2024/06
	Internal control	*IC*	Dibo Internal Control Index of Listed Companies in China/100	_		DIB(https://www.dibcn.com/index.php)	
Control variables	Firm size	*SIZE*	Ln (total assets/10000)	_	(2008–2022) Enterprise-level	Wind (https://www.wind.com.cn)	2024/06
	Leverage ratio	*LEV*	Total debt/Total assets	%			
	Firm age	*AGE*	Ln (Current year of operation – Year of establishment +1)	_			
	Firm age squared	*AGE2*	*AGE* Squared	_			
	Shareholding concentration	*FIST*	Shareholding ratio of the largest shareholder	%			
	Financing constraints	*FINC*	Absolute value of SA index.	_			
	Proportion of independent directors	*INDR*	(Independent directors/the total number of board members)*100	%			
	Board size	*BSIZ*	Ln (Total number of board members)	_			
	CEO duality	*DUAL*	A dummy assigned a value of 1 if the enterprise’s chairman and general manager are the same person, otherwise 0	_			
	Level of economic development	*PGDP*	Ln (Real GDP/population) (2008 base year)	_	(2008–2022) Urban-level	China City Statistical Yearbook (2009–2023)	2024/09
	Level of financial development	*FSIZ*	Financial institutions’ deposit and loan balances/GDP	%			
	Time dummies	*Year*	_	_	_	_	

## 4. Results

### 4.1. Descriptive statistics

Table 2 reports the descriptive statistics of the main variables. The mean value of firm growth (GROW) is 0.2132, with a minimum value of −0.2915 and a maximum value of 2.6788. The mean value of climate risk (CRSK) is 0.4718, with a minimum value of 0.2115 and a maximum value of 0.5777.

**Table 2:** Descriptive statistics.

Variable	Obs	Mean	Std. Dev.	Min	Max
***GROW***	33,568	0.2132	0.4212	−0.2915	2.6788
***rGROW***	33,568	0.1439	0.3145	−0.5551	1.5905
***CRSK***	33,568	0.4718	0.0701	0.2115	0.5777
***rCRSK***	33,568	3.8621	0.1741	3.0979	4.6151
***COST***	33,568	−0.7069	0.1778	−1.0128	−0.1585
***rCOST***	33,568	−0.8706	0.1363	−1.3714	−0.4497
***IC***	31,525	6.4482	1.2563	0.0000	8.6662
***rIC***	31,468	0.0509	1.2306	−6.4041	2.2649
***SIZE***	33,568	12.9816	1.3547	6.3669	19.4262
***LEV***	33,568	0.4247	0.2069	0.0506	0.8986
***AGE***	33,568	2.0869	0.9005	0.0000	3.4965
***FIST***	33,568	34.5161	15.0526	8.6000	75.0500
***FINC***	33,568	3.7898	0.2812	1.8050	5.6459
***INDR***	33,568	37.5285	5.3597	30.7692	57.1429
***BSIZ***	33,568	2.1301	0.2018	0.6931	2.8904
***DUAL***	33,568	0.2733	0.4457	0.0000	1.0000
***PGDP***	33,568	1.7695	0.4916	−0.0258	2.6656
***FSIZ***	33,568	3.6462	1.3638	1.7844	7.3843

### 4.2. Baseline results

We estimated Equation (1) using a fixed-effects model with GROW as the dependent variable and progressively increased the control variables. Prior to interpreting the regression results, we assessed potential multicollinearity by calculating the variance inflation factors (VIFs) for all continuous variables in our full model. The mean VIF is 6.59.

The regression results are presented in columns (1)–(5) of Table 3. All regression coefficients of CRSK2 were significantly negative, and all coefficients of CRSK were significantly positive. For instance, in the full model (column(5) of Table 3, the coefficient on *CRSK* is 0.7419 (90% CI: [0.3548, 1.1290]) and the coefficient on *CRSK2* is −0.7363 (90% CI: [−1.2357, −0.2369]). This pattern confirms an inverted U-shaped relationship between CRSK and GROW. Therefore, all results support H1.

**Table 3:** Regression results for H1.

Variables	(1)	(2)	(3)	(4)	(5)
	*GROW*	*GROW*	*GROW*	*GROW*	*GROW*
***CRSK***	0.9754***^a^	0.7369***	0.6950***	0.7033***	0.7419***
	(0.3106)	(0.2425)	(0.2365)	(0.2375)	(0.2353)
***CRSK2***	−0.7677*	−0.6554**	−0.7014**	−0.7116**	−0.7363**
	(0.3988)	(0.3063)	(0.3045)	(0.3056)	(0.3036)
***SIZE***		0.2063***	0.2027***	0.2030***	0.2031***
		(0.0090)	(0.0089)	(0.0091)	(0.0091)
***LEV***		−0.1341***	−0.1410***	−0.1414***	−0.1421***
		(0.0297)	(0.0296)	(0.0296)	(0.0296)
***AGE***		−0.8710***	−0.8960***	−0.8953***	−0.8955***
		(0.0401)	(0.0398)	(0.0398)	(0.0397)
***AGE2***		0.3360***	0.3470***	0.3468***	0.3468***
		(0.0202)	(0.0204)	(0.0204)	(0.0205)
***FIST***			0.0034***	0.0034***	0.0034***
			(0.0005)	(0.0005)	(0.0005)
***FINC***			0.4890***	0.4899***	0.4912***
			(0.0533)	(0.0533)	(0.0535)
***INDR***				−0.0012*	−0.0013*
				(0.0006)	(0.0006)
***BSIZ***				−0.0252	−0.0253
				(0.0238)	(0.0238)
***DUAL***				0.0140**	0.0142**
				(0.0069)	(0.0069)
***PGDP***					−0.0338
					(0.0437)
***FSIZ***					0.0040
					(0.0081)
Observations	33,568	33,568	33,568	33,568	33,568
*R-squared*	0.0825	0.2621	0.2693	0.2695	0.2695
*N*	3,241	3,241	3,241	3,241	3,241
*Inflection point* ^*b*^	0.6353	0.5622	0.4954	0.4942	0.5038

^a^Robust standard errors are in parentheses. ***p < 0.01, **p < 0.05, *p < 0.1.

^b^According to Equation (1), the inflection point is calculated as -β₁/(2 × β₂), where *β₁* and *β₂* represent the coefficients of *CRSK* and *CRSK2*, respectively.

We calculated the inflection point of the U-shape according to the results of benchmark regression (Table 3). The average value of CRSK is 0.4718, indicating that CSRK in most cities falls on the left side of the inflection point. This suggests that, for the majority of cities in our sample, the current level of climate risk remains at a stage where it promotes firm growth.

### 4.3. Endogenous treatment and other robustness tests

To ensure the robustness of our core findings, we pre-specified and implemented a series of tests designed to address the distinct potential concerns. First, to mitigate endogeneity bias potentially arising from measurement error in the composite climate risk index, we employed a 2SLS estimation with valid instrumental variables. Second, to assess the sensitivity of our results to variable measurement, we re-estimated Equation (1) using alternative proxies for both the *CRSK* and *GROW*. Third, we tested the robustness of our statistical inference to different assumptions about error correlation by changing the level at which standard errors are clustered. Finally, to further alleviate concerns regarding reverse causality, we re-ran the analysis using one-period lagged values of the climate risk variables. The results of these pre-specified tests are presented in Table 4 and discussed below.

**Table 4:** Endogeneity treatment and other robustness tests for H1.

	(1)	(2)	(3)	(4)	(5)
	IV^*a*^	rGROW	rCRSK	SID	LAG
Variables	GROW	rGROW	GROW	GROW	GROW
*CRSK*	1.0562***^b^	0.8711***		0.7419***	
	(0.2606)	(0.1850)		(0.2074)	
*CRSK2*	−1.2890***	−0.7724***		−0.7363***	
	(0.3488)	(0.2109)		(0.2692)	
*rCRSK*			0.9319**		
			(0.4083)		
*rCRSK2*			−0.1199**		
			(0.0581)		
*L.CRSK*					0.7355***
					(0.2312)
*L.CRSK2*					−0.7609***
					(0.2637)
**Year FE**	YES	YES	YES	YES	YES
**Enterprise FE**	YES	YES	YES	YES	YES
**Industry FE**	YES	YES	YES	YES	YES
**Control**	YES	YES	YES	YES	YES
***Observations***	29,953	33,568	33,568	33,568	29,977
***R-squared***	0.1146	0.0768	0.2696	0.2695	0.1148
***N***	3,217	3,241	3,241	3,241	3,241
**Inflection point**	0.4097	0.5639	3.8862	0.5038	0.4833

^a^Instrumental variable estimations pass the validity test.

^b^Robust standard errors are in parentheses. *** p < 0.01, ** p < 0.05, * p < 0.1.

#### 4.3.1. Endogenous treatment.

Previous theoretical analyses and empirical tests have shown that CRSK significantly affects the GROW. Conversely, as a micro-variable, it is difficult for GROW to influence the macro-variables of CRSK, making it tricky to establish a bidirectional causal relationship between CRSK and GROW. However, the CRSK derived from a comprehensive indicator system are inevitably subject to measurement errors that may lead to endogeneity. To address this issue, we conducted a two-stage least squares (2SLS) regression analysis to deal with endogeneity.

Following the established approach of Wang et al. [32,57,58], we construct our instrumental variables as the one-period lagged mean of climate risk in other cities within the same year (*L.i*vCRSK) and its quadratic term (*L.ivCRSK2*). Regarding relevance, CRSK and CRSK2 in all cities were subject to measurement errors. Therefore, L.ivCRSK and L.ivCRSK2 were correlated with CRSK and CRSK2. The use of a one-period lag for the IVs preserves this correlation through the persistence of the climate system. Therefore, L.ivCRSK and L.ivCRSK2 satisfied the correlation condition.

In terms of exogeneity, the mean-centring operation dilutes any special associations that may exist between climate risk of other cities and the target city, resulting in outcomes closer to the climate system trend. Although listed companies may have branches in other cities, their major decisions are typically made by their headquarters, and their operational decisions generally follow the headquarters’ unified coordination. Furthermore, employing the one-period lagged value of other cities’ average risk ensures the instrument is pre-determined in time, making reverse causality from firm performance to past regional climate conditions highly implausible. Therefore, L.ivCRSK and L.ivCRSK2 meet the condition of exogeneity.

We employed GROW as the dependent variable and re-estimated Equation (1) using the instrumental variables (IV) method. The Cragg-Donald Wald F-statistic for the weak IV test is 213.471, exceeding the critical value of 7.03 at a 10% error rate. Consequently, L.ivCRSK passed the weak IV test, confirming that L.ivCRSK is a valid instrumental variable. Subsequently, we re-estimated Equation (1) using instrumental variables (IV*).* The IV estimation results, presented in Column (1) of Table 4, show that H1 remains valid after controlling for endogeneity. The coefficient on *CRSK* is 1.0562 (90% CI: [0.6275, 1.4849]) and the coefficient on *CRSK2* is −1.2890 (90% CI: [−1.8628, −0.7152]).

#### 4.3.2. Other robustness tests.

Next, we replaced the dependent variable with rGROW and re-estimated Equation (1) using the fixed effects model; The results are presented in column (2) of Table 4. We also substituted the independent variable with rCRSK and its squared term rCRSK2. After re-estimating Equation (1) with fixed effects, the results are shown in column (3) of Table 4.

We then changed the clustering of standard errors from the city-year level to the firm-year level. The results are presented in column (4) of Table 4. Additionally, we employed one-period lagged values of *CRSK* as explanatory variables to re-estimate Equation (1), with the results displayed in Column (5) of Table 4. Overall, the findings in columns (2) to (5) of Table 4 confirm that the relationship between *CRSK* and *GROW* follows an inverted U-shaped pattern. These results support the validity and robustness of H1. Moreover, we further calculated inflection points that under various robustness testing scenarios (Table 4).

## 5. Mechanism studies

### 5.1. Mechanism analysis

#### 5.1.1. *Climate risk*, *internal control*, *and firm growth.*

Climate risk exerts pressure on managers, shaping their cognitive appraisal of it as either a challenge or a threat. This appraisal, in turn, has an inverted U-shaped effect on the quality of internal controls. When managers perceive climate risk as a challenge, it can stimulate proactive adaptive behaviors [58], motivating them to leverage available resources (e.g., human, capital, technology) to enhance internal control quality and integrate climate risk responses into operational, strategic, and risk management framework [32], which inherently reinforces the internal control environment.

Conversely, if climate risk is viewed as a threat, managers tend to adopt defensive and contractionary strategies. This may involve cutting investments in internal controls to reduce costs, or a diminished capacity and willingness to maintain them due to heightened financial constraints. In such scenarios, managers often prioritise building financial slack to bolster organizational resilience against climatic threats, which can divert critical resources away from sustaining or enhancing internal control systems [32], ultimately leading to a decline in their quality.

Furthermore, robust internal controls contribute significantly to firm growth through several mechanisms. First, they mitigate agency problems by enhancing information transparency, improving oversight and incentive systems, curbing managerial self-serving behaviour, and providing external investors with accurate information, thereby reducing agency costs [59]. Second, they suppress earnings management by monitoring and constraining manipulation of financial reports, which reduces the negative impact of real earnings management and enhances future performance [60]. Third, they alleviate financial constraints. High-quality internal controls improve accounting information quality [61], leading to more favourable audit opinions and enabling investors to better assess risks [32], which ultimately lowers financing costs and eases constraints.

**Fig 4:**
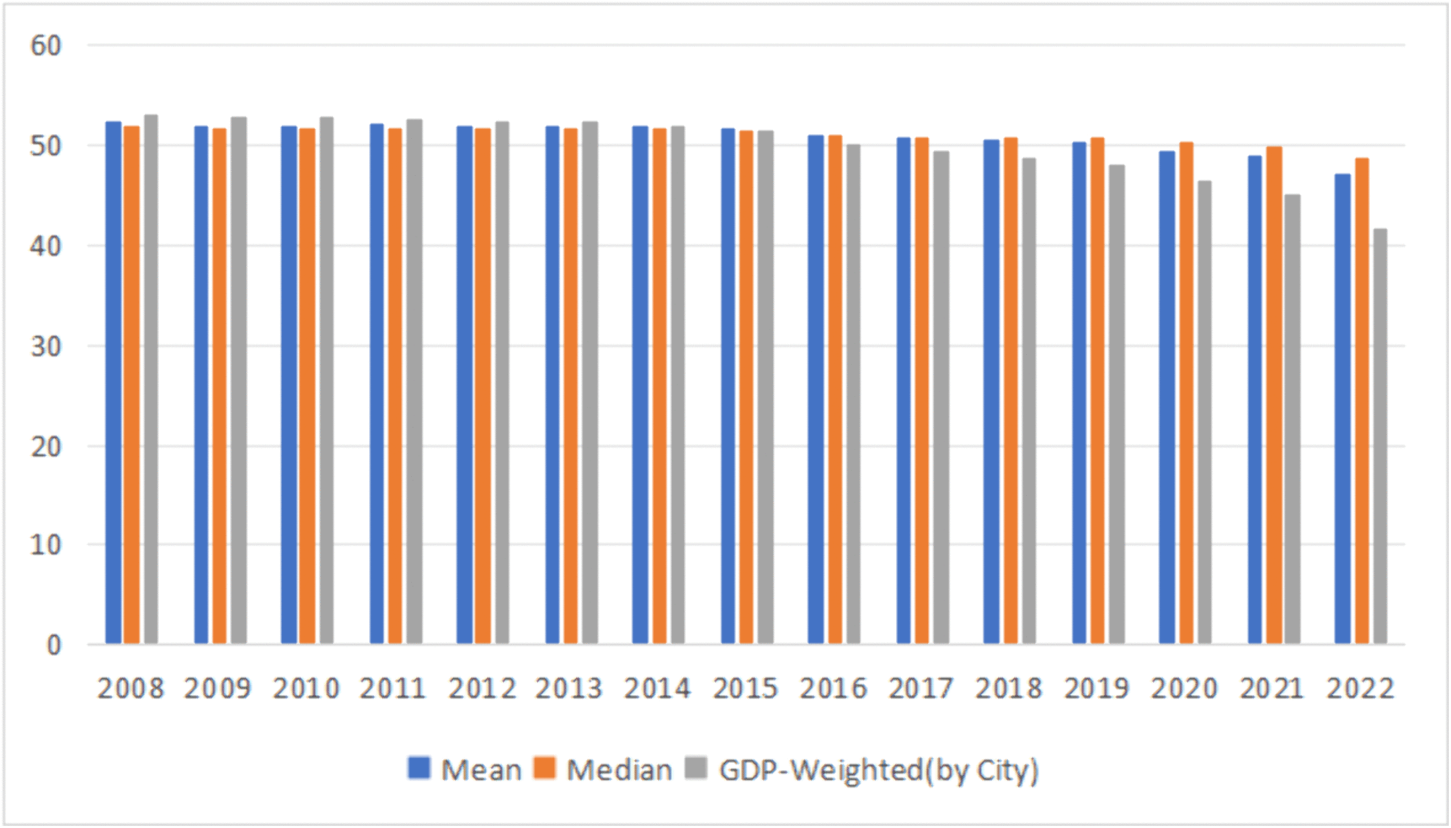
Mechanism path and hypothesis.

We thus propose that (Fig 4)—

H2:Climate risk has an inverted U-shaped effect on firm internal control quality and indirectly exerts an inverted U-shaped non-linear effect on firm growth through its influence on internal control quality.

#### 5.1.2. *Climate risk*, *operating cost control*, *and firm growth.*

Climate risk influences firms’ operating cost control efficiency in an inverted U-shaped manner through managers’ resource assessment logic, which subsequently affects firm growth potential. Drawing on challenge–threat theory, cost control efficiency serves as a key indicator of managers’ operational capability. When managers appraise climate risks as challenges, they are more likely to take proactive measures—such as cutting unnecessary expenditures and optimizing resource allocation—thereby enhancing cost control efficiency. Conversely, if these risks are perceived as threats, cost control may be deprioritised in favour of emergency responses. Managers may retain redundant resources (e.g., excess inventory) as a buffer against uncertainty, leading to deteriorated cost control.

Effective operating cost control, in turn, supports firm growth through two main mechanisms. First, lower operating costs widen profit margins, freeing up resources that can be redirected toward market expansion, R&D, and other growth-enhancing activities. Second, improved cost efficiency reduces credit risk. Cost optimisation boosts net profit and cash flow, thereby strengthening the firm’s debt-servicing capacity and shareholder confidence. This, in turn, mitigates financing constraints and facilitates the growth of firms.

We thus propose that (Fig 4)—

H3: Climate risk has an inverted U-shaped effect on a company’s operating cost control efficiency and indirectly exerts an inverted U-shaped non-linear influence on the firm growth potential through its effect on operating cost control.

### 5.2. Mechanism testing

We designed the following models with reference to Chen et al. [20] for the indirect effects:


$${MEDI}_{it}\;=\;\alpha_{\text{0}}\;+\;\beta_{\text{1}}\times {CRSK}_{it}\;+\;\eta \times Z\;+\;\alpha_{i}\;+\;\lambda_{t}\;+\;\epsilon_{it}$$ (2)

$$GROW\;=\;\alpha_{\text{0}}\;+\;\beta_{\text{1}}\;{\times \;CRSK}_{\text{it}}\;+\;\beta_{\text{2}}\times {CRSK\text{2}}_{it}\;+\;\phi *\;{MEDI}_{it}\;+\;\eta \times X\;+\;\alpha_{i}\;+\;\lambda_{t}\;+\;\epsilon_{it}$$ (3)

In Equations (2) and (3), ${MEDI}_{it}$ is the mediating variable, that is, the level of internal control (IC) or the level of operating cost control (COST) in year *t* for firm i.

#### 5.2.1. Internal control channel.

**Baseline regression.** Following Ni and Guo (2023) [62], internal control (IC) was measured using the “Dibo Internal Control Index of Listed Companies in China”, issued by Shenzhen Dibo Enterprise Risk Management Technology Co. To mitigate the potential halo effects, where better-performing firms tend to receive higher *IC* ratings. We adjusted the *IC* index by regressing it on Return on Equity (*ROE*), while controlling for firm fixed effects. The residuals from this regression represent a performance-adjusted measure of internal control quality, denoted as rIC, which serve as an alternative variable of *IC*.

Using fixed effects models specified in Equations (2) and (3), we tested H2. The results are presented in Table 5, column (1). In Panel A, column (1), the coefficient on *CRSK i*s significantly positive, and that on *CRSK2* is significantly negative, consistent with an inverted U-shaped effect of climate risk on internal control quality. In Panel B, column (1), the coefficient on *IC* is significantly positive, supporting a mediating effect. Meanwhile, *CRSK* remains significantly positive and *CRSK2* significantly negative. Thus, *IC* plays a partial mediating role in the inverted U-shaped relationship between *CRSK* and *GROW,* and H2 holds. This indicates that *CRSK* exerts an inverted U-shaped impact on *GROW (*consistent with H1) by means of its inverted U-shaped effect on *IC*.

**Table 5:** Estimation results of H2 and robustness tests.

Panel A	(1)	(2)	(3)	(4)	(5)
Variables	*IC*	*IC*	*IC*	*rIC*	*IC*
*CRSK*	1.5437**^a^	2.8575***		1.4118**	1.5437***
	(0.6216)	(0.8094)		(0.5947)	(0.5562)
*CRSK2*	−1.4958*	−2.9151***		−1.3584*	−1.4958**
	(0.8143)	(1.1145)		(0.7843)	(0.7188)
*rCRSK*			2.1552*		
			(1.1659)		
*rCRSK2*			−0.2777*		
			(0.1662)		
**Enterprise FE**	YES	YES	YES	YES	YES
**Year FE**	YES	YES	YES	YES	YES
**Control**	YES	YES	YES	YES	YES
**Observations**	31,525	29,947	31,525	31,468	31,525
***R-squared***	0.0689	0.0664	0.0690	0.0679	0.0689
***N***	3,241	3,217	3,241	3,240	3,241
**Panel B**					
	(1)	(2)	(3)	(4)	(5)
**Variables**	*GROW*	*GROW*	*rGROW*	*GROW*	*GROW*
*CRSK*	0.4764***	0.9313***	0.7944***		0.5011***
	(0.1800)	(0.2572)	(0.1733)		(0.1828)
*CRSK2*	−0.5443**	−1.1451***	−0.7079***		−0.5734**
	(0.2420)	(0.3447)	(0.1975)		(0.2457)
*IC*	0.0310***	0.0397***	0.0464***	0.0310***	
	(0.0021)	(0.0023)	(0.0021)	(0.0021)	
*rCRSK*				0.6616**	
				(0.3318)	
*rCRSK2*				−0.0886*	
				(0.0473)	
*rIC*					0.0300***
					(0.0021)
**Enterprise FE**	YES	YES	YES	YES	YES
**Year FE**	YES	YES	YES	YES	YES
**Control**	YES	YES	YES	YES	YES
**Observations**	31,525	29,947	31,525	31,525	31,468
***R-squared***	0.1140	0.1293	0.1020	0.1140	0.1128
***N***	3,241	3,217	3,241	3,241	3,240
***Inflection point***	0.4376	0.4066	0.5611	3.7336	0.4370

^*a*^The instrumental variables all passed validity tests. Robust standard errors in parentheses. *** p < 0.01, ** p < 0.05, * p < 0.1

**Endogenous treatment.** For the same reasons as in Section 4.3, we employ ivCRSK as an instrumental variable and re-estimate Equation (2), with the results presented in Column (2) of Table 5 Panel A. When estimating Equation (3), we considered that higher levels of internal controls can improve a firm’s growth performance, and improved growth performance can strengthen their internal control quality. Thus, a bidirectional causal relationship exists between a firm’s growth and its internal control quality. We followed the approach in section 4.1.3 to calculate the average internal control index of other firms in the same year, yielding ivIC. We then used ivIC along with ivCRSK and ivCRSK2 as instrumental variables and re-estimated Equation (3). Column (2) of Panel B of Table 5 presents the results. From column (2) of Panels A and B in Table 5, after controlling for endogeneity, H2 holds.

**Other robustness tests.** We replaced the independent variable CRSK with rCRSK and the mediator variable IC with rIC and re-estimated Equation (2). The results are shown in Table 5, Panel A columns (3)–(4). We then changed the clustering of standard errors from the city-year level to the firm-year level. The results, presented in Panel A column (5) of Table 5. We subsequently replaced the GROW with rGROW, the CRSK with rCRSK, and the mediating variable IC with rIC, before re-estimating Equation (3). The results are presented in Table 5, Panel B, columns (3)–(5). As shown in Columns (3)–(5) of Table 5, Panels A and B, H2 remains valid.

#### 5.2.2. Operating cost control channel.

Baseline regression. We generated COST according to the negative of (operating costs/operating revenues) as a proxy variable for operating cost control (𝐂𝐎𝐒𝐓). At the same time, we took the negative of ‘(𝐨𝐩𝐞𝐫𝐚𝐭𝐢𝐧𝐠 𝐜𝐨𝐬𝐭𝐬+𝐦𝐚𝐧𝐚𝐠𝐞𝐦𝐞𝐧𝐭 𝐜𝐨𝐬𝐭𝐬+𝐬𝐚𝐥𝐞𝐬 𝐞𝐱𝐩𝐞𝐧𝐬𝐞𝐬)/𝐨𝐩𝐞𝐫𝐚𝐭𝐢𝐧𝐠 𝐫𝐞𝐯𝐞𝐧𝐮𝐞𝐬’ to obtain 𝐫𝐂𝐎𝐒𝐓 as a replacement variable for subsequent robustness testing. We then employed fixed-effects models based on Equations (2) and (3) to test H3. The results, presented in Column (1) of Table 6, indicate that *COST* partially mediates the inverted U-shaped relationship between *CRSK* and *GROW*, with all relevant coefficients statistically significant. These findings support H3.

**Table 6:** Estimation results of H3 and robustness tests.

Panel A	(1)	(2)	(3)	(4)	(5)
Variables	*COST*	*COST*	*COST*	*rCOST*	*COST*
*CRSK*	0.2870***^a^	0.4602***		0.2364***	0.2870***
	(0.0629)	(0.0893)		(0.0576)	(0.0554)
*CRSK2*	−0.2889***	−0.5253***		−0.2771***	−0.2889***
	(0.0764)	(0.1243)		(0.0692)	(0.0701)
*rCRSK*			0.2044*		
			(0.1061)		
*rCRSK2*			−0.0253*		
			(0.0151)		
**Enterprise FE**	YES	YES	YES	YES	YES
**Year FE**	YES	YES	YES	YES	YES
**Control**	YES	YES	YES	YES	YES
**Observations**	33,568	29,953	33,568	33,568	33,568
***R-squared***	0.1510	0.1406	0.1505	0.1667	0.1510
***N***	3,241	3,217	3,241	3,241	3,241
**Panel B**					
	(1)	(2)	(3)	(4)	(5)
**Variables**	*GROW*	*GROW*	*rGROW*	*GROW*	*GROW*
*CRSK*	0.6105**	0.8366***	0.7923***		0.5938**
	(0.2395)	(0.2529)	(0.1804)		(0.2333)
*CRSK2*	−0.6008*	−1.0333***	−0.6912***		−0.5657*
	(0.3076)	(0.3365)	(0.2030)		(0.3037)
*COST*	0.5150***	0.5369***	0.3086***	0.5157***	
	(0.0307)	(0.0360)	(0.0321)	(0.0306)	
*rCRSK*				0.8469**	
				(0.4111)	
*rCRSK2*				−0.1093*	
				(0.0583)	
*rCOST*	0.6105**	0.8366***	0.7923***		0.5938**
	(0.2395)	(0.2529)	(0.1804)		(0.2333)
**Enterprise FE**	YES	YES	YES	YES	YES
**Year FE**	YES	YES	YES	YES	YES
**Control**	YES	YES	YES	YES	YES
**Observations**	33,568	29,953	33,568	33,568	33,568
***R-squared***	0.2788	0.1252	0.0828	0.2788	0.2836
***N***	3,241	3,217	3,241	3,241	3,241
**Inflection point**	0.5081	0.4048	0.5731	3.8742	0.5248

^*a*^Robust standard errors in parentheses. *** p < 0.01, ** p < 0.05, * p < 0.1

**Endogenous treatment and other robustness tests.** Following the order outlined in section 5.2.1, we estimated Equations (2) and (3) with the mediating variable (COST) to test H3. As Table 6 shows, H3 is supported, and the result is robust.

## 6. Heterogeneity test for financial development

According to the challenge–threat theory, an individual’s assessment of stress depends on the degree of alignment between resource needs and availability. The level of financial development directly influences the availability of resources for firms to address climate risk, potentially leading to heterogeneity in the inverted U-shaped relationship between climate risk and firm growth. Therefore, this section systematically analyses the heterogeneous characteristics of the effect of climate risk on firm growth from the perspective of financial development levels.

Financial development moderates the inverted U-shaped relationship between climate risk and firm growth by influencing the availability of financial resources for firms. First, in financially developed regions, banks and other financial institutions have more mature climate risk assessments, more flexible credit resource allocations, and fewer financing constraints. Despite rising climate risks, financial institutions in financially developed regions have stronger risk-bearing capacities, smaller credit contraction magnitudes, and fewer barriers to accessing financial resources. According to the challenge–threat theory, when firms have higher resource accessibility, managers perceive climate risk as a challenge for a longer period. This long-term perception of climate risk as a challenge, rather than a threat, elevates the inverted U-shaped inflection point between climate risk and firm growth in financially developed regions.

We conducted a heterogeneity analysis based on regional financial development levels using the fixed effects estimation Equation (1), and the results are shown in Table 7. First, for regions with different financial development levels, the inverted U-shaped effect of climate risk on firm growth is significant (consistent with H1), and the inflection points for climate risk at different financial levels are calculated accordingly (Table 7). Second, the inflection points of the inverted U-shaped relationship between climate risk and firm growth in financially developed regions are significantly higher than that in less-developed regions. This finding indicates that, in financially developed regions, climate risk can sustainably enhance firm growth. The empirical results confirm the hypothesis that the effect of climate risk on firm growth is financially heterogeneous.

**Table 7:** Heterogeneity test of financial development levels.

	(1)	(2)	(3)
Variables	*GROW*	*rGROW*	*GROW*
***CRSK*** (***high***)	1.3096***^a^	0.8989***	
	(0.3157)	(0.2864)	
***CRSK*** (***low***)	1.8707***	1.4796***	
	(0.4700)	(0.5013)	
***CRSK2*** (***high***)	−1.5008***	−0.8190**	
	(0.4171)	(0.3561)	
***CRSK2*** (***low***)	−2.4554***	−1.8147***	
	(0.6391)	(0.6614)	
***rCRSK*** (***high***)			0.8536**
			(0.4110)
***rCRSK*** (***low***)			1.0133**
			(0.4036)
***rCRSK2*** (***high***)			−0.1087*
			(0.0586)
***rCRSK2*** (***low***)			−0.1467**
			(0.0577)
**Observations**	33,568	33,568	33,568
***R-squared***	0.2698	0.0767	0.2696
***N***	3,241	3,241	3,241
***Inflection point (high)***	0.4363	0.5488	3.9264
***Inflection point (low)***	0.3809	0.4077	3.4536

^a^Robust standard errors are in parentheses. *** p < 0.01, ** p < 0.05, * p < 0.1.

Existing research on climate risks to the financial system generally concludes that climate risks increase risk exposure, lead to higher bad debt losses, and constrict credit channels, thereby exerting negative effects on the financial system [63]. The heterogeneity analysis further captures the differentiated impact of climate risks on enterprises at varying levels of financial development, broadly aligning with the conclusions of Zhang et al. [64,65]. It validates and refines the mainstream literature’s perspective on the negative effects of climate risks, such as expansion of financial system risk exposure and contraction of credit [63,66]. Meanwhile, it provides empirical support for the climate risk opportunities mentioned in a minority of studies [67].

## 7. Discussion and conclusion

Based on data from Chinese listed companies from 2008 to 2022, this study draws the following conclusions using a two-way fixed effects model: (1) Climate risk has an inverted U-shaped relationship with firm growth. Moderate climate risk promotes firm growth by stimulating adaptive strategies. However, after exceeding the threshold value, resource constraints intensify, leading to a significant decline in firm growth. (2) Climate risk affects the quality of internal controls and the level of operating cost controls through the assessment of managers’ challenge or threat perception, which indirectly contributes to firm growth by lowering agency costs, improving surplus quality, and compressing cost space. (3) The level of financial development regulates the location of the inflection point of climate risk, and firms in developed regions exert a more persistent positive impact on amplifying climate risk owing to the higher availability of financial resources. Managers’ perceptions of challenges are more persistent and the growth-promoting effect of climate risk is long lasting. These findings align with emerging international evidence on the complex, non-linear consequences of climate risk. For example, recent research shows that while climate risk disclosure generally enhances firm value, it can turn negative under heightened market attention [68].

The mechanistic study finds that internal control and operating cost control are important pathways through which climate risk affects firm growth. While the existing literature focuses on the contribution of internal control to firm value, few studies link it to climate risk. Our study reveals that climate risk indirectly affects IC and COST by triggering managers’ assessment of challenge or threat perceptions, which in turn contributes to firm growth. This finding expands the boundaries of the study of firm growth drivers and compensates for the neglect of non-financial indicators in the traditional literature.

In addition, this study finds that the level of financial development significantly moderates the location of the inflection point of climate risk and that firms in developed regions can perceive climate risk as a challenge in the longer term owing to the higher accessibility of financial resources, deepening the theoretical content of regional heterogeneity studies and providing a basis for differentiated policy design.

Based on these findings, we believe that firms should dynamically assess their climate risk levels. During periods dominated by challenges, they should increase technological upgrades and optimise capital allocation and internal control systems to enhance their resource buffering capabilities. During periods dominated by threats, they should prioritise the retention of highly skilled talent, reasonably allocate capital to avoid redundant expenditures, and protect core technological resources to avoid falling into a negative cycle. Simultaneously, companies should improve their early warning systems for climate risk and formulate differentiated support policies for different regions.

This study has certain limitations. First, the measurement of climate risk may not be sufficiently comprehensive. Future research could develop separate indicators for physical risk and transition risk, and examine their respective effects on firm growth. Second, in analyzing the impact of the macro-level variable *CRSK* on the micro-level variable *GROW,* the data matching is based on the climate risk indicators of the city where a company’s headquarters is located, without accounting for the climate risks exposure of its subsidiaries due to data availability constraints. Subsequent studies could broaden the scope of data collection to include climate risk information from the cities where subsidiaries operate, thereby correcting this bias.

## Supporting information

S1 Table Composition and description of *CRSK* indicators. (DOCX)

S2 Table Explained variance ratio. (DOCX)
