# Supplementary material for: How does the climate risk affect the firm growth: Evidence from China
Source: PLoS One. 2026 Feb 25;21(2):e0343426. doi: 10.1371/journal.pone.0343426 (PMC12935275; doi:10.1371/journal.pone.0343426)
Supplement: S1 Table — (DOCX) [file pone.0343426.s001.docx]

## **S1 Table. Composition and Desc**ription of *CRSK* Indicators.

| Indicators | Unit | Coverage | Data Sources | Positive/Negative nature | Access date |
| --- | --- | --- | --- | --- | --- |
| Carbon Dioxide Emissions | Ton | (2008-2022)  Urban-level | China Carbon Accounting Database (https: //www.ceads.net.cn/) | + | 2024/07 |
| Sulphur Dioxide Emissions |  |  | China Urban Statistical Yearbook | + |  |
| Industrial Dust Emissions |  |  |  | + |  |
| Total Number of Enterprises | Unit |  | Financial China Information & Technology Co., Ltd. (https: //www.qyyjt.cn/) | + |  |
| Total Registered Capital of Enterprises | CNY |  |  | + |  |
| Number of Climate-Friendly Technology Patent Applications | Unit |  | Patsnap, whose data originates from the China National Intellectual Property Administration (https: //www.zhihuiya.com) | — |  |
| Proportion of Climate-Friendly Technology Patent Applications to Total Patent Applications | % |  |  | — |  |
| Number of climate-friendly technology patents granted | Unit |  |  | — |  |
| Proportion of climate-friendly technology patent grants relative to total patent grants | % |  |  | — |  |
| Air quality excellence rate | % |  | China Urban Statistical Yearbook | — |  |
