# Supplementary material for: How does the climate risk affect the firm growth: Evidence from China
Source: PLoS One. 2026 Feb 25;21(2):e0343426. doi: 10.1371/journal.pone.0343426 (PMC12935275; doi:10.1371/journal.pone.0343426)
Supplement: S2 Table — (DOCX) [file pone.0343426.s002.docx]

**S2 Table. Explained Variance Ratio.**

| Factor | Eigenvalue | Variance Contribution Rate | Cumulative Contribution Rate |
| --- | --- | --- | --- |
| Factor1 | 3.45619 | 0.3456 | 0.3456 |
| Factor2 | 1.62353 | 0.1624 | 0.5080 |
| Factor3 | 1.48654 | 0.1487 | 0.6566 |
| Factor4 | 1.00891 | 0.1009 | 0.7575 |
